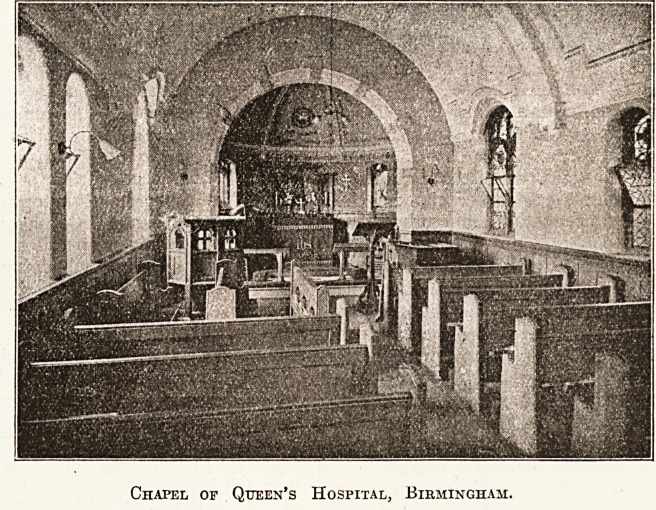# Some Provincial Hospital Chapels

**Published:** 1915-01-30

**Authors:** 


### II.—SOME PROVINCIAL HOSPITAL CHAPELS.

### QUEEN'S HOSPITAL, BIRMINGHAM.

The Queen's Hospital Chapel is a beautiful little modern structure, erected at the last extension of the hospital. It was built from designs by Mr. Ewen Harper, and the chancel was consecrated by Bishop Gore in 1898, who also, when Bishop of Birmingham, made a gift of the altar-rail. The chapel is rectangular, and the chancel is in the form of an apse. The pulpit and seats are made of oak, as also is the reredos, with carved figures of Our Lord, the Blessed Virgin, and St. John. The altar, its ornaments, and all the decorations and additions to the chapel are gifts from various friends. The sanctuary was richly embellished by Mr. William Kermeth, of Edgbaston, as a personal tribute to the thirty years' work of his friend, Mr. Arthur Hulme, the deceased superintendent and secretary. The students of the Birmingham Technical School have made and presented many gifts, among them being the lectern, the work of the carpenters, the cross and altar book-rest, executed by the brassworkers, and the font—a unique specimen of lead-work—contributed by the plumbers. The large Bible and pulpit antependium were presented by Mr. and Mrs. Hulme, and the altar-linen and book-markers by the nurses and their friends, while the stained-glass work is the gift of Alderman Clayton and Messrs. Jones and Willis. There is also a beautiful stained-glass window which was removed from the former chapel, once situated at the end of what is now Jordan Lloyd Ward, and another window is shortly to be placed in the chapel as a memorial to the work of the doctors and nurses.

The chaplaincy is endowed with £40 a year, and there is also a small endowment to provide for an organist and choir, the endowment of the chaplaincy being founded in 1840 through the benefaction of Dr. Sands Cox.

The chapel will hold seventy persons, and on May Day there is an especially beautiful service on the Roof Ward of the hospital. The Chaplain is the Rev. G. H. Moore, who has held the office for the past five years. He has recently written the following special hospital hymn, dedicated to the Matron, Sisters, and Nurses of the Queen's Hospital, Birmingham :

*A Chaplain's Hymn.* (Tune, St. Anne.)

O God, to Thee with perfect trust
We turn our eyes and pray.
We feel that Thou alone canst rule
And guide our earthly way.

That Thou alone canst trace the chart
Of life's uncertain sea,
And safely guide frail human souls
To heavenly destiny.

That Thou alone canst raise the veil
'Twixt us and Thee that stands,
And show that constant help is near
And safety in Thy hands.

Lord, open Thou our eyes that we
May see Thine angels near,
And feel whate'er the future holds
Our hearts need have no fear.

To Thee, O Lord, with perfect trust
Our life, our all we tend,
In fervent hope, life's sorrows passed,
Before Thy throne to bend.

(*To be continued.*)

Chapel of Queen's Hospital, Birmingham.

## Figures and Tables

**Figure f1:**